# Enhanced external counterpulsation treatment improves multi-organ hemodynamics for postoperative liver transplantation patient. A case report

**DOI:** 10.1186/s13019-024-02783-y

**Published:** 2024-05-10

**Authors:** Xinchen Zeng, Xin Jin, Zi’an Wu, Jun Hu, Wenjuan Zhou, Xuelian Shen, Jianhang Du

**Affiliations:** 1 Department of Liver Surgery, The Third People’s Hospital of Shenzhen, Shenzhen, 518112 China; 2 Medical Research Center, The Eighth Affiliated Hospital of Sun Yat-sen University, Shenzhen, 518033 China; 3 School of Biomedical Engineering, Sun Yat-sen University, Shenzhen, 518107 China; 4 Department of Cardiology, The Eighth Affiliated Hospital of Sun Yat-sen University, Shenzhen, 518003 China; 5 Department of Ultrasound Medicine, The Eighth Affiliated Hospital of Sun Yat-sen University, Shenzhen, 518003 China; 6 National Health Commission (NHC), Key Laboratory of Assisted Circulation, Sun Yat-sen University, Guangzhou, 510080 China

**Keywords:** Liver transplantation, Ischemic symptoms, External counterpulsation, Rehabilitation

## Abstract

**Introduction:**

Post liver transplantation (LT) patients endure high morbidity rate of multi-organ ischemic symptoms following reperfusion. We hypothesize that enhanced external counterpulsation (EECP) as a typical non-invasive assisted circulation procedure, which can efficiently inhibit the relative ischemic symptoms via the systemic improvement of hemodynamics.

**Case presentation:**

A 51-year-old male patient, 76 kg, 172 cm, received orthotopic LT surgery for viral hepatitis B induced acute-on-chronic liver failure hepatic failure. His medical records revealed ischemic symptoms in multi-organ at the time of hospital discharge, including headache, refractory insomnia, abdominal paralysis, and lower limb pain. The EECP treatment was introduced for assisted rehabilitation and to improve the postoperative quality of life. Doppler Ultrasound examination showed significant augmentation of blood flow volume in the carotid arteries, the hepatic artery, the portal vein and the femoral artery during EECP intervention. A standard 35-hour EECP treatment led to significant improvement in quality of life, e.g. sleep quality and walking ability.

**Conclusion:**

We report a case of multi-organ ischemic symptoms in a post LT patient. EECP treatment can significantly improve the quality of life via the systematic promotion of hemodynamics.

## Introduction

Liver transplantation (LT) is the standard curative option for end-stage liver diseases and hepatic malignancies [1]. However, successful long-term transplant outcomes are limited by multiple risk factors, especially ischemia reperfusion injury (IRI) [2]. IRI may potentially lead to not only primary graft dysfunction or non-function, but also systemic cardiovascular implications and even multi-organ failure [3, 4], which represents one of the major threats to survival rate and quality of life post LT procedure.

Considering that normal pharmacological treatment such as the vasopressors is not always sufficient, the use of mechanical circulatory support (MCS) such as intra-aortic balloon pump counterpulsation (IABP) has been employed to locally improve the hemodynamics and stabilize the patient post LT in recent years [5]. However, IABP is a relative costly invasive procedure with risks of severe adverse events (e.g. bleeding and major limb ischemia) [6]. Therefore, to develop non-pharmacological and non-surgical rehabilitation approaches are necessary for post LT patient to improve hemodynamic environment and to reduce the risk of ischemic symptoms. Modern external counterpulsation (EECP) might potentially be a feasible option [7].

EECP is a U.S. Food and Drug Administration (FDA)-approved non-invasive assisted-circulation technique originally derived from our laboratory, which involves application of three sets of pneumatic cuffs wrapped around the calves, lower thighs, and upper thighs of the patient, with sequential inflations and deflations synchronized with electrocardiograms [8]. As the result, retrograde blood flow in the aorta, venous return and cardiac output are significantly increased during EECP intervention [9], as well as the systemic blood flow augmentation in arterial segments. EECP exhibits similar but to some extent better hemodynamic effects in contrast to IABP [10], and has been recommended as a clinical therapeutic option for treatment of ischemic cardiovascular and cerebrovascular diseases [11].

Based on the above background, we hypothesize EECP to be an efficiency, safe and low-cost rehabilitation therapy for post LT patients enduring ischemic symptom, especially multiple organs ischemic symptoms. Our current study aims to carry out a perspective study to validate the acute and chronic outcomes.

## Case presentation

A 51-year-old Chinese male patient of 76 kg, 172 cm and a BMI value of 25.69, who underwent orthotopic LT surgery for viral hepatitis B induced acute-on-chronic liver failure hepatic failure in our hospital on February 16, 2021. After the surgy, the patient accepted standard medicine procedure of anti-hepatitis B viral (Propofol Tenofovir tablet), anti-CMV (Valganciclovir), anti-infection (Meropenem combined with Teicoplanin, Caspofungin and Ganciclovir), anti-rejection (Tacrolimus combined with Mycophenol sodium and Methylprednisolone), libile drugs, renal function protection, and venous nutritional support. Meanwhile, an intermittent hemodialysis treatment was performed to improve IRI induced renal inadequacy with a very high serum creatinine level (529.6 µmol/L). The patient was discharged on March 24, 2021. After that, the patient followed medicine procedure like the above with regular follow-up examination every two months.

**Fig. 1:**
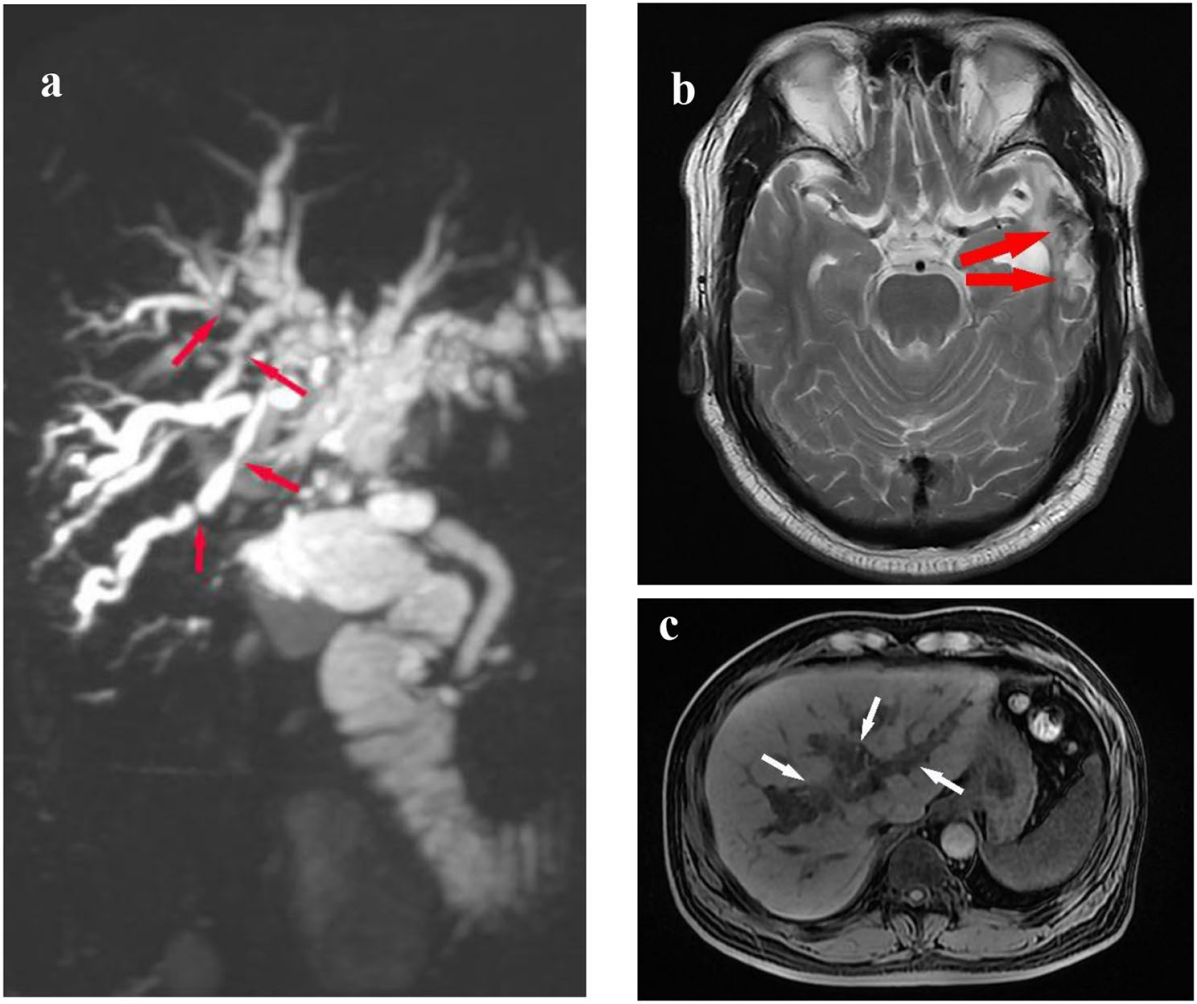
Imaging findings one year after the surge. (**a**) Magnetic Resonance Cholangiopancreatography (MRCP) scan showing ischemia-induced interrupted biliary stenosis, marked with red arrows. (**b**) MRA brain scan showing cerebral edema at the right, marked with red arrows. (**c**) MRA scan of the abdomen showing ischemia-induced cholangiectasis, marked with white arrows

The medical records revealed that the patient claimed successive postoperative complications in multiple organs and relatively poor quality of life successively about half year after the discharge, which mainly involved headache and severe insomnia, abdominal paralysis, lower limb pain and dysbasia. The Pittsburgh sleep quality index (PSQI) test at six months after the discharge showed a score of 14, which indicated the severe sleep disorder. The number of getting up in one night were more than three. Computed tomography angiogram (CTA) scan showed no significant structural lesions in intracranial or hepatic arteries, except for the suture sites. Color Doppler ultrasound examination showed no significant atherosclerotic plaques in carotid or lower extremity arteries. The 6-min walk distance (6MWD) test was not conducted, considering that the patient claimed significant pain in the lower limbs and express reluctance. Color Doppler ultrasound scan in the lower limbs showed a relatively poor blood supply in the common femoral vein of both sides. Blood biochemical test showed a high serum total cholesterol of 5.53 and a high triglyceride of 5.51. Medical imaging examinations showed cerebral edema as well as ischemia-induced interrupted biliary stenosis and cholangiectasis (see Fig. 1).

We diagnosed these to be IRI-related chronic ischemic symptoms. However, half-year conventional medicine treatments could not improve these symptoms significantly. Meanwhile, the patient could not afford active exercise-based rehabilitation treatments such as cardiopulmonary exercise or daily physical exercises. Under this circumstance, the traditional EECP as a noninvasive assisted circulatory and passive exercise technique was introduced in this case to systematically improve the hemodynamics and ischemic symptoms in multiple organs.

**Fig. 2:**
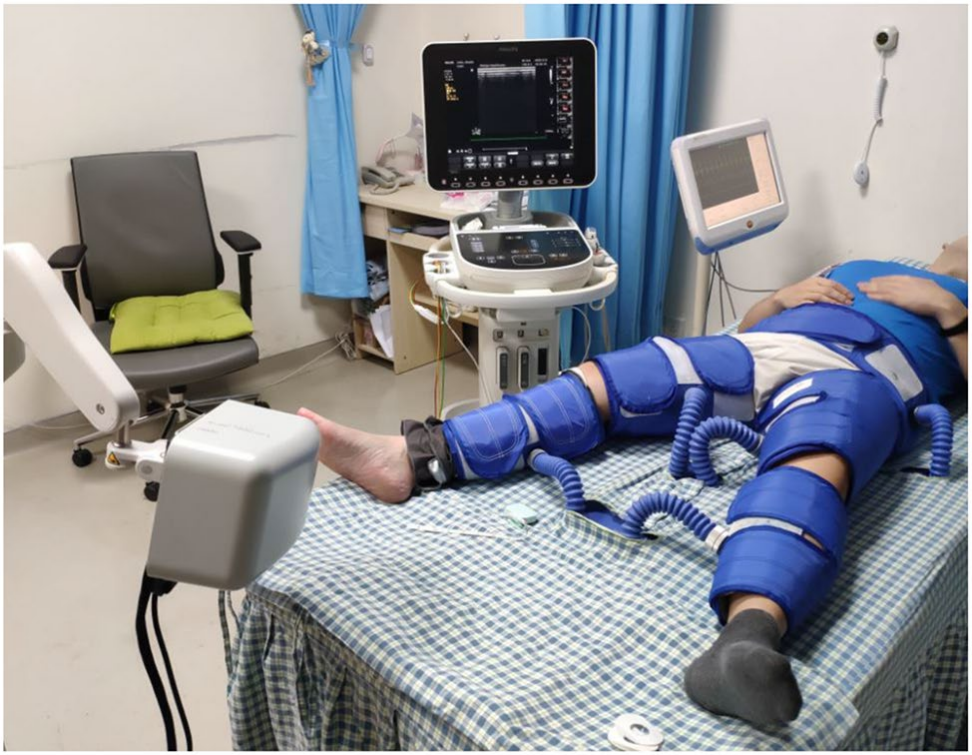
EECP treatment and ultrasound measurement

The patient underwent a standard EECP treatment from April 22 to June 25 in 2022, which involved a one-hour daily programme, 5 days a week, for a total of 35 sessions of 7 weeks (See Fig. 2). However, a relatively low EECP treatment pressure of 30 kPa was applied in this case for safety concern, comparing to the normally setting of 35 to 40 kPa for the treatment of coronary heart disease and angina.

**Fig. 3:**
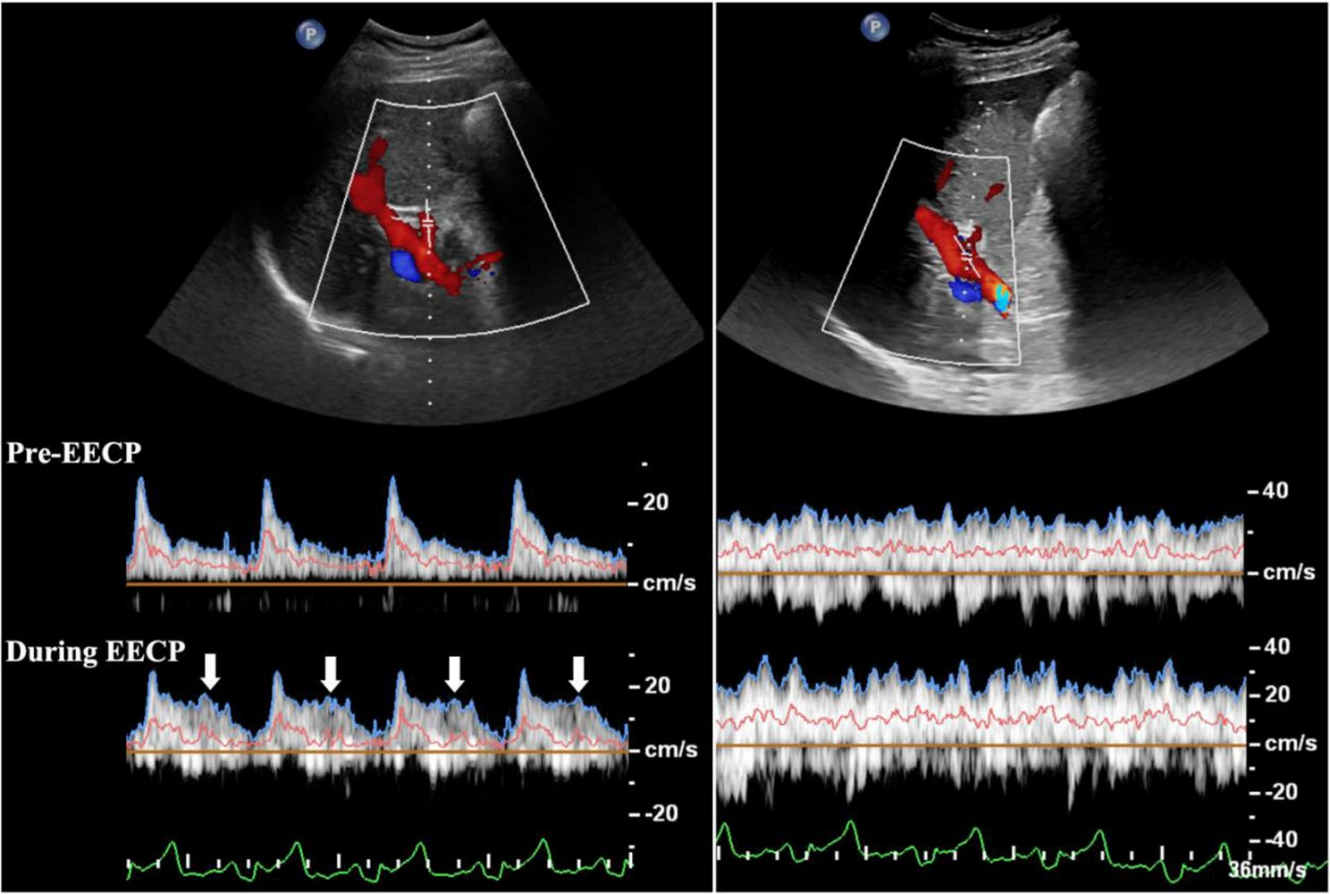
Ultrasound flow velocity spectrums in the proper hepatic artery (left) and portal vein (right). The blue, pink, and green curves represent the peak flow velocity waveforms, mean flow velocity waveforms, and ECGs, respectively. White arrows indicate the diastolic augmentation induced by EECP stimulation

The blood flow velocity spectrum variations within the proper hepatic artery (PHA) and portal vein (PV) pre and during EECP intervention are showed in Fig. 3, which were measured in four continuous cardiac cycles based on a color Doppler ultrasound system (EPIQ7C, Philip Com, Netherlands) equipped with a 9–11 MHz multifrequency high-resolution linear probe. The results indicate that EECP apparently changed the blood flow pattern in the PHA and led to a significant augmentation of the diastolic blood flow velocity. Table 1 shows the variations of the hemodynamic parameters in four vascular segments, which include the peak systolic velocity (PSV), mid- diastolic velocity (MDV), mean flow rate (MFR) over the cardiac cycle, antegrade and retrograde volume flow over one cardiac cycle.

**Table 1:** Hemodynamic variations pre and during EECP treatment

	PHA		PV		CCA		CFA	
	Pre	During	Pre	During	Pre	During	Pre	During
Diameter, mm	6.4	6.6	14.0	14.1	7.3	7.8	8.8	9.1
PSV, cm/s	26.63	27.29	31.20	34.75	81.89	84.27	105.49	345.06
MDV, cm/s	8.46	15.34	25.46	30.44	38.69	66.50	2.54	-272.32
MFR, ml/s	3.70	4.32	19.14	20.68	16.81	23.62	7.11	17.95
antegrade volume flow, ml	1.48	1.86	15.31	17.78	6.72	10.16	5.58	26.39
retrograde volume flow, ml	-------	-------	---------	----------	--------	----------	2.8	18.64

After 10-hour EECP treatment, the patient claimed a noticeable improvement in sleep quality. The insomnia symptoms improves and the number of getting up in the night reduced. After the fully 35-hour EECP treatment, the patient’s quality of life significantly improved compared to the condition prior EECP treatment. The PSQI test showed a score of 7 and the number of getting up in one night reduced to 0 continuously. The patient could afford a continuous outdoor walk over an hour, as well as can climbing stairs on a daily basis. Imaging and biochemical examination showed good function of both the liver and the kidney.

After the standard 36-h EECP treatment, the patient returned to the conventional medicine procedure. Six months after the EECP treatment, the patient claimed a slight lower limb pain and applied for a second EECP treatment. But no abdominal paralysis was claimed and the sleep quality remained good.

## Discussion

To improve the hemodynamic circumstance and the perfusion of the ischemic organs and tissue play the key role for the treatment of IRI related symptoms in postoperative LT patients [5]. Our current study introduces EECP as a noninvasive assisted circulatory therapy to improve the IRI related mult-organ symptoms in postoperative LT patients via the continuous and systematic improvement of hemodynamic environment. Instant hemodynamic analysis based on ultrasound measurement indicates that EECP intervention slightly increases the lumen diameters within the four vascular segments of PHA, PV, CCA, and CFA, compared with the pre-EECP condition. Accordingly, the PSV level increases 2.5% in the PHA, 11.38% in the PV, 2.91% in the CCA, and 227.10% in the CFA, respectively. The MDV level increases 81.32% in the PHA, 19.60% in the PV, and 71.88% in the CCA, respectively. In the CFA, the sequential distal to-proximal inflations of the three sets of EECP pneumatic cuffs led to a rapid turn of the antegrade blood flow in the systole and a retrograde MDV of 272.32 cm/s compared with the antegrade MDV of 2.54 cm/s pre-EECP. The MFR level increases 16.76% in the PHA, 8.05% in the PV, 40.51% in the CCA, and 152.46% in the CFA, respectively. The volume flow over the cardiac cycle increases 25.68% in the PHA, 16.13% in the PV, and 51.19% in the CCA, respectively. In the CFA, EECP intervention augments 372.94% of the antegrade volume flow and 565.71% of the retrograde volume flow over the cardiac cycle. During EECP treatment, heart rate (HR) of the patient showed a plateau compared with the pre-EECP condition, which suggests that EECP intervention causes no increase in the heart burden. Similar results were confirmed in previous studies of healthy volunteers and patients with cardiovascular disease [11, 12].

Meanwhile, a standard 35-h EECP treatment significantly improved the quality of life of the patient and promoted the rehabilitation. The patient claimed a significant improvement of sleep quality after the long-term EECP treatment. PSQI test showed a score of 7 compared to 14 before the treatment. Abdominal paralysis completely disappeared which revealed the improvement of local microcirculation. Lower limb pain completely disappeared. The dysbasia was significantly improved and the walking ability increased.

## Conclusions

We report the application of EECP as a noninvasive rehabilitation therapy in a LT patient who suffered from multi-organ ischemic symptoms after the surge. Standard 35-h EECP treatment effectively improved the ischemic symptoms and promoted the quality of life, via the continuous improvement of the systemic hemodynamic environment. We suggest that EECP may be a safe, efficient and low-cost rehabilitation strategy for postoperative LT patient with IRI related symptoms, especially for those who are unsuitable for physical exercises and refractory to conventional pharmacological treatment.

## Data Availability

The datasets used and/or analysed during the current study are available from the corresponding author on reasonable request.
