# Correction: Enhanced external counterpulsation treatment improves multi-organ hemodynamics for postoperative liver transplantation patient. A case report

**DOI:** 10.1186/s13019-024-03118-7

**Published:** 2024-11-01

**Authors:** Xinchen Zeng, Xin Jin, Zi’an Wu, Jun Hu, Wenjuan Zhou, Xuelian Shen, Jianhang Du

**Affiliations:** 1 Department of Liver Surgery, The Third People’s Hospital of Shenzhen, Shenzhen, 518112 China; 2 Medical Research Center, The Eighth Affiliated Hospital of Sun Yat-Sen University, Shenzhen, 518033 China; 3 School of Biomedical Engineering, Sun Yat-Sen University, Shenzhen, 518107 China; 4 Department of Cardiology, The Eighth Affiliated Hospital of Sun Yat-Sen University, Shenzhen, 518003 China; 5 Department of Ultrasound Medicine, The Eighth Affiliated Hospital of Sun Yat-Sen University, Shenzhen, 518003 China; 6 National Health Commission (NHC), Key Laboratory of Assisted Circulation, Sun Yat-Sen University, Guangzhou, 510080 China

**Correction: Journal of Cardiothoracic Surgery (2024) 19:284.** 10.1186/s13019-024-02783-y

Following publication of the original article [1], a new funding information section needs to include in this article and should have read 'Shenzhen High-level Hospital Construction Fund (XKJS-PWK-001)'.

Corrected Funding Section:

Funding

This work is supported by Science and Technology Planning Project of Shenzhen Municipality (Grant No. JCYJ20230807111059040), Public Health Key Project of Futian District of Shenzhen (FTWS2021001) and Shenzhen High-level Hospital Construction Fund (XKJS-PWK-001).

The original article has been corrected.
